# Using the Collective Impact Model to Organize Evidence-Based Programs to Educate Health Care Professionals About the Care Needs of Individuals with Intellectual and/or Developmental Disabilities

**DOI:** 10.3390/healthcare14152338

**Published:** 2026-08-01

**Authors:** Sarah H. Ailey, Dianne Cooney Miner, Suzanne C. Smeltzer, Beth Marks, Jasmina Sisirak, Brian Abery, Renata Tichá

**Affiliations:** 1 Department of Community, Systems, and Mental Health Nursing, Rush University College of Nursing, Rush University Medical Center, Chicago, IL 60612, USA; 2 Golisano Institute for Developmental Disability Nursing, St. John Fisher College, Rochester, NY 14618, USA; dcooneyminer@childrensinstitute.net; 3 Children’s Institute, Rochester, NY 14604, USA; 4 M. Louise Fitzpatrick College of Nursing, Villanova University, Villanova, PA 19085, USA; 5 Department of Disability and Human Development, University of Illinois Chicago, Chicago, IL 60608, USA; bmarks1@uic.edu (B.M.);; 6 Institute on Community Integration, University of Minnesota, Minneapolis, MN 55414, USA

**Keywords:** intellectual disabilities, developmental disabilities, health care professionals, education, program development, Collective Impact Model

## Abstract

**Background:** Individuals with intellectual and/or developmental disabilities (IDDs) experience persistent health inequities, exacerbated by the systemic lack of education of health care professionals about their care. In response to a 2020 call from the Administration for Community Living in the United States, five institutions formed the IDD Health Equity Consortium to develop a suite of educational materials and practice experiences to improve the education of health care professionals in the health and health care of individuals with IDDs. **Methods:** The Collective Impact Model, designed to align organizations and stakeholders around a shared agenda for system change, was used to organize IDD Health Equity Consortium programs. Backbone infrastructure included a cross-sector steering committee, an Advocate Advisory Committee, and three Consortium Action Networks focused on communication, measurement, and education, practice, and policy. A scoping review of the literature was conducted, and a Participatory Planning and Decision-Making process engaged individuals with IDDs, family members, students, faculty, and professionals in identifying important themes for developed materials. **Results:** Learning modules, case studies, service-learning experiences, and simulation experiences were developed across Consortium institutions and were disseminated across multiple other institutions, with beginning alignment with interprofessional and disability competencies. Mixed-methods evaluation strategies assessed learner outcomes, including knowledge checks and pre and post evaluations using established measures of skills, comfort levels, and approach; and interprofessional socialization, valuing, and collaborative practice behaviors. **Conclusions:** By involving individuals with IDDs in curriculum development and building multi-institution and cross-sector infrastructure, the Consortium developed scalable materials that address longstanding gaps in health care professional education. The developed suite of educational materials and practice experiences and early evaluation findings provided a foundation for further program refinement and future empirical studies examining long-term effects on practice and clinical outcomes.

## 1. Introduction

Worldwide, an estimated 107.6 million individuals (≈1.74% of the population) have intellectual disabilities [1], and 240–290 million children have developmental disabilities [2]. Individuals with intellectual and/or developmental disabilities (IDDs) are active and valued members of their families and communities, participating in employment, education, leisure, and religious life. Despite this, they continue to experience significant health disparities, including poorer health outcomes across the lifespan, higher rates of chronic conditions, and shorter life expectancies than the general population, patterns that reflect persistent negative social determinants of health, the non-medical, environmental, and social conditions that directly influence a wide range of health outcomes [3]. Health outcomes are worse for individuals with IDDs who have intersecting identities as members of racial, ethnic and other groups facing systemic discrimination [4,5,6].

Addressing these inequities would require a health care workforce prepared to deliver high-quality, person-centered care to individuals with IDDs. International and national initiatives, including the United Nations Convention on the Rights of Persons with Disabilities [7], the World Health Organization’s Global Disability Action Plan [8], and the United States (US) National Council on Disability’s Health Equity Framework [9], have called for integrating disability-related content into health-professional education. In the US, the Alliance for Disability in Health Care Education (ADHCE), with support from the Centers for Disease Control and Prevention, developed the Core Competencies on Disability for Health Care Education (Core Competencies) to establish baseline expectations for training health care professionals [10,11].

Emerging evidence also suggested that tailored hospital programs for patients with IDDs, including structured staff education, may improve system-level outcomes. Hospitals without such programs were indicated to incur higher costs and significantly longer lengths of stay, particularly for patients with IDDs who had extreme high acuity levels [12]. These findings underscored the potential impact of workforce preparation on measurable health outcomes.

Despite these initiatives, IDD-inclusive education for health care professionals remains limited, often confined to single institutions or isolated efforts. Most US health care professional accreditation organizations have not required disability-specific (including IDD-specific) content [9], and efforts to introduce disability-focused content frequently have met administrative and faculty resistance to adopting non-required and unfamiliar materials [11]. These gaps are further exacerbated by the continued influence of the medical model of disability, which shapes health care professional training in ways that perpetuate bias, limit meaningful patient and family engagement, and inadequately prepares a health care workforce that can provide the full continuum of care across the lifespan for individuals with IDDs [13,14].

To address these gaps, the US Administration for Community Living issued a 2020 call for proposals to build a national consortium aimed at embedding research-informed content on the health care needs of individuals with IDDs into health care professional education in a scalable and nationally significant manner [15].

In response, and pre-program, faculty from five institutions with long-standing collaboration in IDD-inclusive health care education formed the IDD Health Equity Consortium (Consortium; also known as PATH-PWIDD (Partnering to Transform Health Outcomes with Persons with IDD) [15]. The Consortium’s goal was to develop a suite of educational materials and practice experiences to improve the education of health care professionals in the health and health care of individuals with IDD. The partnering institutions developed programs for the suite of materials that were intentionally designed to fit within existing educational structures without requiring major curricular changes. This strategy was mean to allow institutions to integrate content in ways that aligned with their structures, resources, and readiness for change. Materials were also meant to be able to meet future educational standards and accreditation expectations when these occur.

This article describes the development, implementation, and early-stage outcomes of the IDD Health Equity Program.

## 2. Materials and Methods

### 2.1. Description of Consortium

The IDD Health Equity Consortium was formed with five institutions. Institution One, a public university in a Midwestern city, has housed an IDD and community inclusion center since 1985 [16] with deep expertise in disability advocacy and community-based supports [17,18]. Institution One was home to the state’s federally designated University Center for Excellence in Developmental Disabilities (UCEDD) [19] and the Leadership Education in Neurodevelopmental and Related Disabilities (LEND) program [20]. Institution Two, a private university with an academic medical center in a second Midwestern city, has had an Americans with Disabilities Act Task Force since 1991 [21] and a committee to improve the care of individuals with IDDs since 2007 [22]. It has developed multiple education programs, many involving student projects in their development, to improve care with individuals with IDDs [23] and has implemented a robust interprofessional education course featuring virtual wellness-goal development with older adults [24]. Its pre-licensure nursing program also includes community-based clinical experiences with organizations serving individuals with IDDs [25]. Institution Three, a public university with an academic medical center in the same city, has had a university department on disability and human development with a disability studies program since 1997 [26], and has brought extensive experience in health promotion with a long-standing program launched in 1998 [27] to improve health outcomes and access to health care, promotion of disability-informed ethics, and training of professionals and the community in advocacy in disability rights and policy [28,29]. Like Institution One, it also housed the state’s federally designated UCEDD and it was part of the state’s LEND program [19,20]. For both Institution One and Three, being part of these long-standing US federal programs brought interprofessional training capacity, policy expertise, and national leadership in IDD programs and community engagement to the Consortium. Institution Three also had a simulation lab and interprofessional learning environments. Institution Four, a private university in the northeastern United States, had developed, in 2018, a fellowship for nursing thought leaders in the care of individuals with IDDs [30], had integrated IDD-focused content and practice experiences into its pre-licensure nursing curriculum [31], and had developed an early iteration of the Advanced Practice Providers Residency program in the care of individuals with IDD [32]. Institution Five, a private university in a mid-Atlantic state, had, since 2015, integrated disability-focused content into its undergraduate and graduate nursing curricula and had developed simulation experiences involving individuals with disabilities [33].

Faculty leading the Consortium represented extensive expertise in IDD health education and practice, including two past presidents of the Alliance for Disability in Health Care Education, developers of established health-promotion programs for individuals with IDDs, a director of an institute for IDD nursing thought leaders (and a former dean of a school of nursing), and experts in community integration and disability advocacy. This collective expertise was further strengthened through cross-sector collaboration with advocates with IDDs, family members, community-based workforce partners, accreditation and licensing specialists, and health economists, ensuring that program development was informed by research, practice, policy, and lived experience.

### 2.2. Program Framework

The lack of education of health care professionals in the health and health care of individuals with IDDs is a complex issue requiring coordinated action across sectors and stakeholders, including individuals with IDDs, their families, educators, clinicians, policymakers, and organizations positioned to influence systemic change. Considering the complexity of the issues, the Consortium used the Collective Impact Model [34] for development, implementation and dissemination of a suite of educational materials and practice experiences in the care of individuals with IDDs. The Collective Impact Model emerged in response to concerns about fragmented and uncoordinated approaches to social-change initiatives [34]. It has been applied across multiple large-scale efforts in which diverse stakeholder groups work together to address complex persistent problems, such as obesity, diabetes, and community health disparities, through development of interventions with coordinated strategies aligned around a common agenda [35].

The Collective Impact Model comprised five phases: generating ideas and hosting dialog, initiating action, organizing for impact, beginning implementation, and sustaining action and impact; and five core tenets: common agenda, backbone support, mutually reinforcing activities, shared measurement, and continuous communication. The phases and tenets provided a structured framework for multi-institution and cross-sector collaboration and guided decision making in the development of the Consortium programs.

The use of the Collective Impact Model was not without concerns. Early formulations did not explicitly incorporate an equity lens or emphasize engagement of affected communities [36,37]. Subsequent critiques highlighted challenges related to power imbalances, limited mechanisms for ensuring meaningful participation of marginalized groups, and inadequate attention to sustaining backbone infrastructure in ways that support shared leadership among participating organizations [38,39]. Efforts have been made to strengthen implementation of the model [40]. Achieving shared measurement across diverse institutions was also considered difficult [34]. These concerns informed the Consortium’s approach and shaped the equity-focused strategies described below. The phases, program years and months, and activities aligned with tenets of the Collective Impact Model are described below and in Table 1.

#### 2.2.1. Phase One: Generating Ideas and Hosting Dialog

Generating ideas and hosting dialog included formation of the IDD Heath Equity Consortium and the application for and obtainment of Administration for Community Living funding. These activities assisted with development of the tenet of common agenda.

#### 2.2.2. Phase Two: Initiating Action

In order to initiate action in the development of a suite of educational materials and practice experiences and related programming, Consortium leadership organized training of Consortium staff and supporters by FSG in Collective Impact, organized initial discussions on intersectionality, conducted a scoping review of the literature to gain common understanding of existing efforts to educate health care professionals in the care of individuals with IDDs, and conducted a Participatory Planning and Decision-Making Process to determine themes to guide the program.

##### Training of Staff

To support the development of a common agenda, in year one (September 2020–August 2021), leaders and staff from the five partner institutions (PI, Co-Is, program coordinators, leaders of action networks, and other leaders at partner institutions) and advocates as a group (n ≈ 30) completed training in the Collective Impact Model available through FSG, a global Foundation Strategy Group that provided customized consultation about large-scale social issues change through use of Collective Impact [41]. Hosted by partners at Institution Four, located in a small city with a history of initiatives using the Collective Impact Model [42,43], training in the model was held over two meetings in January 2021 (year one). About half of participants were from Institution Four. The PI and all co-Is from the five consortium partners, at least one advocate, and leaders interested in establishing consortium action networks from a few other institutions were in attendance. Later in year one, to address intersectionality [4,5,6], Consortium leaders collectively discussed the literature on intersectionality, including Black Lives Matter: A commentary on Racism and Public Health [44] and Race and Health Disparities in Adults with Intellectual and Developmental Disabilities Living in the United States [45], attended by about 15 people from the five Consortium partner institutions.

In year two, Consortium leaders completed training with the National Center for Interprofessional Practice and Education concentrated on building synergy and dissemination of our work. The training included a “Laying the Groundwork” session on interprofessional education and practice (IPE) program development and a case study exercise that helped participants examine macro-, meso-, and micro-system factors influencing IPE planning across the five core partner institutions. Leaders were able to discuss institution-specific considerations. Also in year two, Consortium leaders completed training in health advocacy hosted by Institution Three. The training focused on connecting advocacy with addressing social determinants of health, ableism, and culturally accessible health care for individuals with disability, including individuals with IDDs, as a key component of equitable health care [46]. Each of these trainings included approximately 15 people from across the five partner institutions.

##### Scoping Review

To establish a rigorous evidence base for program design, Consortium leaders and students from Institutions One and Two conducted a comprehensive scoping review. Planning began in year one. The purpose was to examine (1) the state of education since 1990 preparing health care students and professionals to address the health and health care needs of individuals with IDDs, and (2) trends and progress over that period. The 1990 start date reflected the passage of the Americans with Disabilities Act, an internationally significant milestone in disability rights [47]. Guided by the Framework for Educating Health Professionals to Address the Social Determinants of Health [48], the team, supported by a research librarian, searched PubMed/MEDLINE, Scopus, CINAHL, PsycINFO, and ERIC using keywords related to health profession education, IDDs, interprofessional training, and discipline-specific education. The search, conducted in March 2022 (year two), yielded 4948 articles, with 182 meeting inclusion criteria after screening in Covidence. Data extraction in Qualtrics captured publication characteristics, disability and interprofessional models, competency-based approaches, involvement of individuals with IDDs or families, and evaluation methods, which informed the final analytic categories. Reports on progress of the scoping review were made to the Cross-Sector Steering committee and Advocate Advisory Committee. Authors included an interprofessional team of faculty (nursing, occupational therapy, special education, psychology), a Ph.D. student, and a LEND fellow. Other faculty, graduate students, and LEND fellows were acknowledged. The team included members with lived experience of disability [47].

The scoping review identified several critical gaps in existing educational programs. Most programs were not informed by overall health care workforce needs to address the care of individuals with IDDs across the continuum of care and lifespan; were not based on a specifically non-medical model of disability such as a social or civil rights model; did not address intersectionality; and were not developed with or evaluated by individuals with IDDs and/or family members [47]. Most programs were single site without efforts for dissemination.

National bodies had emphasized that evaluating outcomes for community members, not only learners, was a critical priority [48]. However, outcome evaluations of the effect of educational programs for health care professionals on individuals with IDDs were rare. When reported, they were limited, such as small, non-significant improvements in oral-health indicators following staff training [49] and basic reporting of screening and referral rates in student-led clinical experiences [50].

Student self-evaluations of their knowledge, skills, and attitude development were the most common means of evaluation. Identifying themes from student reflections were the most common method of qualitative evaluation. In some cases, evaluation only consisted of explanations that programs were feasible and acceptable to users. The review also identified positive trends aligned with the Framework, including use of competency-based approaches, interprofessional education, and experiential learning in many reviewed programs [47].

##### Participatory Planning and Decision-Making Process

Using findings from the scoping review and based on participant experience, partners from a community integration program at Institution One led a Participatory Planning and Decision-Making (PPDM) process at the beginning of year three to develop themes that would guide the work of the Consortium. The PPDM was a model-building approach grounded in participatory action research and involved structured steps such as pre-surveys, facilitated discussions, and iterative refinement of themes [17,51,52]. The partners at Institution One had previously applied the PPDM to develop validated frameworks in areas such as self-determination, health care coordination, and home- and community-based services for youth and adults with IDD [51,53,54]. Using convenience sampling through contacts from the Advocate Advisory Committee, Consortium Action Networks, and implementing institutions, 22 participants, including individuals with IDDs, family members, students, professionals, and faculty, engaged in the PPDM process [55].

Before initial meetings, participants completed pre-surveys or interviews reflecting on themes from the scoping reviews. Each group then first met separately to identify themes from the scoping review and their own experiences that they viewed as essential to developing the Consortium’s programs, followed by a joint meeting.

Using nominal group procedures [56], facilitators guided the process and supported participants in reaching consensus on the themes identified via the pre-survey. The facilitators guided participants to weigh identified themes by their importance on a scale from 0 to 100 or 0 to 10 (individuals with IDDs) using PPDM software, followed by a discussion leading to consensus (agreement higher than 80%). In the discussion, participants explained their weightings, could adjust their scores based on what they were hearing from others, and could also add themes or subthemes that may have been missing. The identified themes included the following: (1) the need for dedicated interprofessional education in the care of individuals with IDDs; (2) critique of the medical model of disability and attention to social, civil rights, and other inclusive models of disability; (3) attention to intersectionality; (4) meaningful involvement of individuals with IDDs in program design, implementation, and evaluation; and (5) the use of experiential learning approaches.

While the PPDM participant sample may not have represented the full range of perspectives across racial, ethnic, linguistic, and disability backgrounds, the lived experiences of individuals with IDDs were reflected, along with family, student, faculty, and professional viewpoints from different regions of the United States. This process helped bring forward priorities from members of the IDD community and informed program development beyond solely professional perspectives. Collectively, the initiatives assisted Consortium partners in developing a common agenda.

#### 2.2.3. Phase 3 Organizing for Impact

Infrastructure and the tenet of backbone support were central to organizing for impact and preparing to implement the suite of educational and practice experiences and other programming. Critiques of the Collective Impact Model, particularly its early lack of attention to risks of over-centralized leadership and power imbalances, and its limited attention to engagement of affected communities, informed the Consortium’s intentional approach to distributing leadership and embedding community engagement throughout development of programs. Developing shared-measurement strategies and mutually reinforcing activities were also essential.

##### Distributing Leadership and Embedding Engagement of Community

Institution Two served as the primary backbone institution responsible for coordinating project infrastructure, convening partners, and supporting cross-sector alignment. To avoid over-centralization, Institution Four led development of a communication protocol and managed the Consortium website and social media platforms starting the first year. To further address potential power imbalances, the Consortium established a Cross-Sector Steering Committee that included Consortium partners, leaders in interprofessional education and IDD-inclusive health care, and advocates. Consortium Action Networks were established in year one. Based on reflections on advocate input gathered through the Cross-Sector Steering Committee and Consortium Action Networks, Consortium leaders recognized that additional opportunities to strengthen shared leadership were needed. In year two, the Advocate Advisory Committee was established as an intentional mechanism to ensure that advocates had meaningful influence over decisions, priorities, and program direction.

All IDD Health Equity Consortium partners needed to establish backbone supports appropriate to their specific programming. These included securing faculty time for teaching, creating course sites within institutional learning management systems, arranging physical or virtual space for service-learning and simulation activities, and ensuring processes to recruit and compensate participating individuals with IDDs, as well as the staff who supported their participation, and family members for their time and contributions to programs.

##### Consortium Action Networks

Three Consortium Action Networks; Communication, Measurement, and Education, Practice, and Policy, were established in year one as collaborative spaces to support the tenet of mutually reinforcing activities. The Consortium Action Networks brought together leaders who were independently developing initiatives related to health care professional education and IPE, measurement including how to measure bias, and communications strategies along with professionals in health care and related fields, faculty, students, family members, and advocates. Leadership of the Consortium Action Networks was shared between faculty from the five partner institutions who worked to determine content and presenters for meetings, with backbone support for schedules and communication from Institutions Two and Four. Consortium Action Networks sessions allowed participants to share work, learn from one another, and build cross-institutional relationships. For example, during years one and two, the Education, Practice & Policy Action Network hosted five presentations from faculty and practitioners leading health care professional training in health care with individuals with IDDs, IPE curricula, and experiential learning programs across the United States. These sessions enabled Consortium partners to learn from national exemplars and to disseminate their own emerging practices. Such sessions continued throughout the grant period. There were approximately 70 participants across the three Consortium Action Networks, contributing to the development of a broad base of mutually reinforcing activities.

##### Advocate Advisory Committee

In year two, an Advocate Advisory Committee, composed of compensated self-advocates and family advocates, was organized. Initial members were largely recruited by Institutions One and Two, with later members recruited from self-advocacy organizations and from individuals known by participants in the Consortium Action Networks. Advocates included individuals from marginalized racial and ethnic communities. While all members spoke English, some also spoke or understood other languages. Beyond discussion of specifically IDD Health Equity programs, others with existing programs or trying to start programs presented their work to the advocates and received advice. Often it was pointed out that discussion had raised one or more issues not considered prior to meetings. Periodic meetings were held to “brainstorm” on what might be missing, what to do next, and/or what additional should be performed. Ideas generated were used and considered important. It was thought that these inputs would not have happened with advocate participation just on the Cross-Sector Steering Committee and the Consortium Action Networks. As such, the Advocate Advisory Committee served as an equity-focused governance structure within the Collective Impact Model. It provided enhanced means for individuals with lived experiences to share leadership and ongoing guidance for Consortium programs and provided leadership opportunities beyond Consortium programs.

##### Infrastructure for Continuous Communication

Early planning identified the need for stronger backbone support to facilitate continuous communication across diverse stakeholder groups in the Consortium programs. In response, a monthly newsletter and website were launched in year one and managed at Institution Four. The original grant supported a part-time content strategist; additional funding at the end of year one enabled full-time content-strategy support.

The content strategist collaborated with the team to create a communication plan informed by implementation science and health literacy principles [57]. Key activities based in content-strategy models included identifying stakeholder groups, tailoring content to their needs, mapping materials to specific audiences, and promoting resources [57]. In addition to publications and conference presentations and posters, the team developed a website and social media strategies to support ongoing engagement and stronger cross-sector alignment with educators, practicing professionals, administrators, policy experts, individuals with IDDs and their families, and other stakeholders.

##### Developing Shared Measurement Strategies

Shared measurement strategies were considered essential for advancing health equity for individuals with IDD, yet they remain underdeveloped across health professional education and health care systems. The Consortium confronted a well-recognized challenge in health professions education: most programs relied heavily on knowledge tests and self-reported confidence or attitudes, which do not evaluate downstream patient outcomes [48]. To build on the expertise of others, beginning at the end of year one, the Consortium’s leadership convened Measurement Action Network presentations featuring experts in IPE outcomes assessment and international outcomes measurement efforts with individuals with IDDs. Three institutions adopted validated instruments: Institutions Two and Four used Interprofessional Education Collaborative (IPEC) Core Competencies [58] assessment tools to evaluate IPE socialization, valuing, and collaborative practice behaviors [59,60]. Institutions Two and Five used Robey’s Self Attributions of Skill, Comfort, and Approach (Self Attributions) Scale to evaluate students’ perceived readiness to provide care to individuals with IDDs [61]. All institutions also employed structured student reflections as a qualitative measure of learning and professional growth. The issue of measuring downstream outcomes remained a problem.

##### Developing Mutually Reinforcing Activities

In the Collective Impact Model, mutually reinforcing activities referred to coordinated actions undertaken by different organizations or stakeholders that are aligned with a shared vision and common agenda but are distinct and complementary within a coordinated strategy [34]. Programs developed at each institution drew on existing strengths and prior initiatives, with strengths and resources varying across the partner institutions. For example, Institutions Three and Five had established simulation centers with experience in providing simulation to interprofessional students, and Institution Five had prior experience engaging individuals with disabilities as simulated patients [33], enabling rapid development of simulation-based learning experiences with individuals with IDDs. Institution Two built on an existing service-learning experience embedded in a required IPE course [24] and long-term existing relationships with organizations serving individuals with IDDs [25] for the development of a service-learning experience with individuals with IDDs. Institution Four leveraged its existing networks and efforts to integrate IDD-inclusive care into its curriculum [30], launched a fellowship to improve IDD-inclusive health care in nursing [31], and developed an Advanced Practice Providers Residency program [32] to develop new programs. Institutions One and Three contributed deep expertise in disability advocacy, community engagement, and interprofessional training through their UCEDD and LEND programs [19,20].

The partners also recognized the variability among institutions that train the health care workforce (e.g., large public universities, private universities, and small private colleges), all of which were represented in the Consortium. The developed educational materials and practice experiences appropriately varied across partner institutions, with some intentional overlap.

## 3. Implementation of Consortium’s Suite of Educational Materials and Practice Experiences (Phase Four)

**Figure 1:**
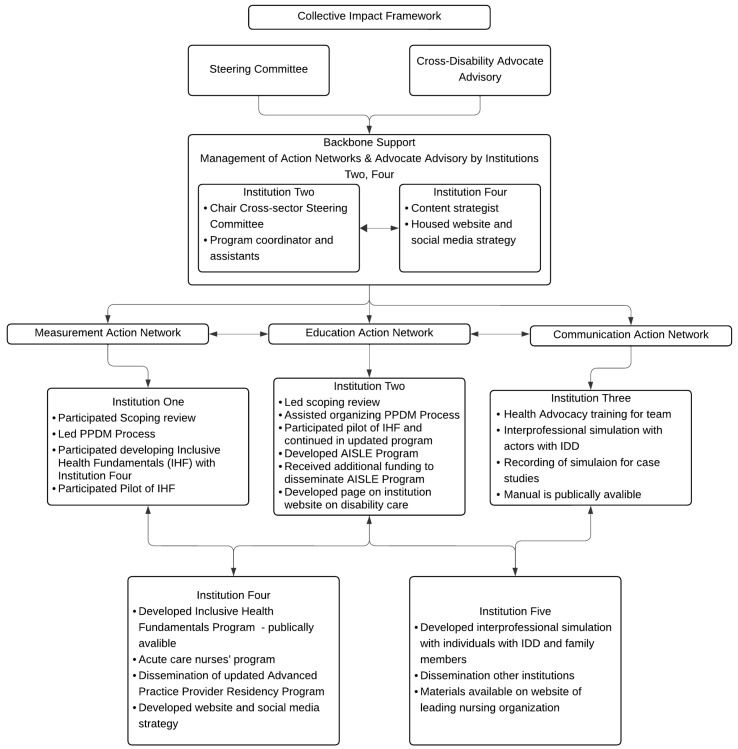
Collective Impact Model structure and IDD Health Equity Consortium partner programs.

This section summarizes the Consortium’s core educational materials and practice experiences that all began implementation in year three. These coordinated efforts reflect the benefit of the Collective Impact Model, showing how shared goals and complementary activities produced programs no single institution could have developed independently [34]. Figure 1 illustrates the Consortium’s backbone structure and partner programs.

### 3.1. Institution One Programs

As noted, Institution One, along with Institution Two, led the scoping review and led the PPDM process. Institution One also contributed to and evaluated the pilot of a program developed at Institution Four, and these efforts are described under Institution Four programs.

### 3.2. Institution Two Programs

Institution Two developed an Advanced Interprofessional Service-Learning Experience (AISLE) and a disability-care webpage. It also participated in piloting the Inclusive Health Fundamentals course (see Institution Four—Section 3.4) with continuation after the pilot.

#### 3.2.1. Advanced Interprofessional Service-Learning Experience (AISLE)

Developed with a community organization serving adults with IDDs in socially vulnerable neighborhoods, AISLE centered addressing intersectionality and structural inequities [62]. To participate in AISLE, students needed to have completed a required interprofessional course [24]. All students at the university were eligible after completion of the required course. Participating students completed a voluntary ten-week program with nine modules on communication, disability models, social determinants of health, bias, telehealth, motivational interviewing, cultural humility, and chronic-condition health promotion. Modules included videos featuring individuals with IDDs and brief knowledge checks. Students received a crosswalk linking content to ADHCE core competencies [10] and IPEC competencies [58]. Interprofessional student teams conducted three virtual telehealth sessions with individuals with IDDs, focused on partnering to develop a wellness-goal with action plan with each individual with IDDs [62]. Participating individuals with IDD and staff were compensated with $25 gift cards for participation. Students completed reflections and pre/post evaluations and earned a micro-credential upon completion. The Advocate Advisory Committee reviewed the program.

The virtual telehealth sessions were conducted evenings during the week. Students first sent dates when they were available and asked individuals with IDDs if any of these times were suitable. If not, further efforts were made to find suitable times. Evenings during the week were chosen as students were usually not in classes or clinicals and individuals with IDDs were home from day programs or jobs. Individuals with IDDs and staff were in residential homes and together at one location during the telehealth sessions.

With additional funding [63], AISLE was later adapted to be integrated into community health nursing clinicals at two universities, a pilot of a course for health interprofessional students with a service-learning component at a third university, and into a community-engaged disability-justice course at a fourth university. These partners are currently developing a cross-institutional implementation manual that outlines concrete examples of implementation of AISLE in different contexts and challenges. The manual is meant to provide examples to other interested institutions that can be used when adapting the program for their institutions. The manual is planned to be publicly available.

#### 3.2.2. New Website Resource on Disability Care

Institution Two also created a disability-care webpage with input from faculty, nursing students, and the Advocate Advisory Committee, including accessibility resources, provider lists, and testimonials [64].

### 3.3. Institution Three Program

Institution Three developed in-person and telehealth simulations with trained actors with IDDs to support IPEC competencies [65]. Students completed preparatory modules and participated in telehealth or on-site sessions. In the second year, students also completed health-advocacy training [46]. Modules and simulation activities were mapped to ADHCE Core Competencies [10] and IPEC Core Competencies [58]. The program was disseminated to other institutions including Institution Five for graduate students. A manual for the simulation was developed and is available through Institution Five [66].

### 3.4. Institution Four Programs

Institution Four developed three major workforce-development initiatives.

#### 3.4.1. Special Olympics International Inclusive Health Fundamentals

With Special Olympics funding, Institutions Four and One developed the flexible Special Olympics International Inclusive Health Fundamentals (SOI-IHF) five-module course, which provides foundational content on the health needs of individuals with IDDs for both clinical and non-clinical staff [67]. Institutions One and Two participated in the pilot implementation, and Institution Two has continued to enroll students in the course. Because the SOI-IHF modules were designed to be used either individually or as a complete sequence, other institutions could integrate the content in ways that best aligned with their existing training structures. This enhanced the course’s adaptability and scalability.

#### 3.4.2. Hospital-Based Acute Care Nurses Training Program

With Mother Cabrini Foundation support, Institution Four produced a 13-module, self-paced online series to improve hospital care for individuals with IDDs provided by acute care nurses [68,69]. Modules covered communication, pain assessment, and behavioral and mental health. The program was offered at no cost to an initial 110 acute care nurses and other professionals. Consortium partners at other institutions assisted with development of some of the modules.

#### 3.4.3. Advanced Practice Providers Residency Program Expansion

An earlier primary care residency for nurse practitioners caring for individuals with IDDs was expanded to include family medicine and physician assistant residents [32]. The enhanced Advanced Practice Providers Residency eight-module curriculum added lifespan and behavioral health content and supported system-level improvement efforts [32]. The Advocate Advisory Committee reviewed the program. It has been disseminated to six additional residency programs, with further expansion underway.

### 3.5. Institution Five Program

Institution Five expanded its existing disability-inclusive nursing curriculum through new simulation experiences developed with individuals with IDDs and family members. Interprofessional student teams conducted virtual interviews with individuals with IDDs portraying patients transitioning to new health care practices and participated in debriefings [70].

Students completed seven preparatory modules on communication, disability models, intersectionality, social determinants of health, bias, and common IDD-related health. Emphasis was made on support for individuals with IDDs in their interactions with health care providers in acute or community-based settings, and interprofessional collaboration [70]. The experience was a requirement for nursing students in their senior year at Institutions Five and Occupational and Physical Therapy students in graduate programs at another university. Pre/post evaluation included a knowledge test and Robey’s Self-Attributions Scale [61]. The Advocate Advisory Committee reviewed the program [70].

The simulation model was adopted by additional institutions [70], and information on the simulation program related resources are available through a national nursing organization for nursing education programs, leaders, faculty [71].

## 4. Results

### 4.1. Application of PPDM Themes in Program Development

The Participatory Planning and Decision-Making Process identified five themes that guided program development across the Consortium. Programs developed across the five Consortium institutions reflected these themes. Each program was specifically designed to educate health care professionals in the care of individuals with IDDs and was interprofessional in structure. Programs explicitly addressed models of disability. The Inclusive Health Fundamentals course, the AISLE program, and the preparatory modules for Institution Five’s simulation experience each included dedicated content on the social model of disability [62,67,70]. Institution Three grounded its preparatory materials in a health and disability justice model [65]. The AISLE program and the simulation preparatory materials at Institutions Three and Five explicitly addressed intersectionality [62,65,70]. All programs incorporated content on social determinants of health, bias, cultural and societal barriers to inclusive care, communication strategies, and approaches to supporting self-determination in health care. Meaningful involvement of individuals with IDDs occurred through the Advocate Advisory Committee and through presentations and feedback at Consortium Action Network meetings. Experiential learning was incorporated across programs. Case studies were used in all programs. Institution Two’s AISLE program implemented a service-learning experience [62], and Institutions Three and Five implemented simulation-based experiences [65,70]. The service-learning and simulation experiences included direct interaction with individuals with IDDs.

Consistent with the emergence of competency-based IDD-inclusive education of health care professionals identified in the scoping review [47], programs at Institutions Two, Three, and Five aligned to ADHCE and/or IPEC core competencies.

### 4.2. Pre–Post Evaluation of Programs

#### 4.2.1. Institution One Evaluations

Institution One was involved in development of programs at Institution Four and related evaluation are discussed in Institution Four evaluations.

#### 4.2.2. Institution Two Evaluations

Across eight AISLE terms, 161 students from 11 programs completed 148 telehealth sessions with 55 Community Health Mentors (89.7% of expected). Fewer than 10% of eligible students participated. The student cohort was racially and ethnically diverse (55% White; 45% students of color, including 20% Hispanic, 20% Black, and 19% Asian); fewer than 10% identified as having a disability, approximately 80% identified as female, and about half were nursing students [72]. AISLE was implemented through a community-based organization serving neighborhoods that were 70–90% Black. Among individuals with IDDs partnering with students, more than 90% identified as Black (60% male, 40% female). Staff supporting participants were predominantly female and more than 90% Black [62,72].

A pre–post evaluation across eight terms used Robey’s Self-Attributions Scale [61] and the Interprofessional Socialization and Valuing Scale-9 (ISVS-9) [59]. Students created anonymous five-digit IDs to link responses. Students, faculty, individuals with IDDs, and staff also completed the combined Telehealth Competency Questionnaire–Provider (TCQ-P) [73] and Interpersonal Process of Care (IPC) Survey [74]. Significant improvements were observed. ISVS-9 scores increased from 50.5 to 55.1, t(92) = 5.705, *p* < 0.001, 95% CI [2.99, 6.18] (d = 0.59). Robey’s Self-Attributions scores decreased from 62.9 to 41.9, t(92) = 9.344, *p* < 0.001, 95% CI [16.51, 25.43] (d = 0.97), indicating improved skill, confidence, and comfort. Missing data were not imputed; student data without matched pre/post data were excluded listwise.

Individuals with IDDs, staff, students, and faculty completing the TCQ-P/IPC survey generally reported clear communication, respectful engagement, and effective collaboration on wellness goals. Some noted occasional issues with rapid speech or overly complex language [62,72]. Completion of evaluation measures was voluntary, and not all students completed both assessments.

#### 4.2.3. Institution Three Evaluations

At Institution Three, student collaborative practice behaviors of the fall 2022 cohort of nurse practitioners, dental, and pharmacy students involved in the interprofessional simulation experience involving individuals with IDDs as actors were evaluated through mixed methods. Pre–post improvements across all domains of interprofessional collaborative practice behaviors using the IPEC SET 27 [60] were found (n = 68, *p* < 0.0001) [65]. Profession-specific differences were observed. The greatest gains reported in values and ethics were among pharmacy students, roles and responsibilities and communication among dental students, and teamwork among nurse practitioner students. Qualitative debriefings reinforced these findings, highlighting increased confidence, role clarity, and readiness to apply collaborative behaviors when caring for underserved populations, including individuals with intellectual and developmental disabilities [65].

#### 4.2.4. Institution Four Evaluations

Evaluation of the pilot of the five-module Inclusive Health Fundamentals course was conducted at Institution One. A total of 586 students participated, with 441 students completing a pre-evaluation and 412 a post-evaluation. Students were 96.8% female and 79.1% nursing students; 51.7% had completed some previous training in health of individuals with IDDs and 88% were aged 18–25. In pre-evaluation, about 44% reported being moderately to very personally knowledgeable about IDDs, shifting to about 65% post-program (*X*^2^ = 38.0, *p* < 0.01); and 65% being moderately to very confident in communication with individuals with IDDs, shifting to about 92% post-program (*X*^2^ = 90.1, *p* < 0.01) [75]. The program was then updated and placed in a learning management system. It had a modular structure designed for flexible use, allowing institutions to implement some or all modules. A 2024 Annual Report indicated that 124 universities were using the course and that 29,176 clinicians and students had engaged with the SOI-IHF and other virtual education in 2024 [76].

Evaluation of the acute care nurse training program (n = 110 nurses) demonstrated improvements. At baseline, 26% of learners reported above-average or very high knowledge levels, compared with 88% post-training (target = 80%). Objective knowledge scores increased from 67% to 74% (*p* = 0.2—not significant). Confidence in caring for patients with IDDs rose from 27% at baseline to 86% post-completion (target = 80%; *X*^2^ = 78.3, *p* < 0.01). Nearly all learners (99%) reported that the content influenced their thinking or approach to care, and all participants indicated they would change their practice because of the program. At the time of evaluation, 90% of learners who had already cared for individuals with IDDs post program (n = 24) reported that the training positively influenced their comfort, confidence, perceived patient outcomes, and patient satisfaction [77].

The Advanced Practice Provider Residency program demonstrated a similarly strong impact. All participants reported increased knowledge, competence, and comfort in caring for individuals with IDDs, and 95% anticipated greater clinical confidence and reduced stress in future encounters. Participants emphasized that the curriculum enhanced broader clinical skills including communication, bias recognition, and clinical reasoning and that the material was immediately applicable to practice. Notably, 76% of participants reported feeling prepared to implement organizational improvements, including changes to workflow, communication practices, accessibility, and staff preparation [78].

#### 4.2.5. Institution Five Evaluations

For Institution Five, reported evaluation was based on 153 nursing, occupational therapy, physical therapy, pharmacy, and physician assistant program students, of whom 99 completed pre and post surveys [70]. Students were 72.2% female and 27.3% male, with about 2/3 being nursing students and 1/3 being occupational and physical therapy students. Evaluation further reinforced Consortium-wide trends. Scores on Robey’s Self-Attributions Scale improved (t ≈ 7.27, *p* < 0.001; independent *t*-test). Reflections highlighted shifts in perceptions and readiness for practice; students noted that the experience helped “break stereotypes” of what you think about someone with IDDs and helped “prepare for an encounter with someone with IDD” [70]. The reflections aligned with quantitative improvements in skill, comfort, and approach.

#### 4.2.6. Limitations of Evaluations

For Institution Five, simulation-based evaluation results were presented at a conference. For Institution Three, the simulation-based evaluation results were published in a peer-reviewed manuscript. Confidence intervals and effect sizes were not reported at the conference nor in the manuscript. For evaluation of pre–post data for the SOI-IHF [75] and for the simulation experiences at Institution Five [70], potential reasons for attrition were not described in presentations about the programs.

### 4.3. Shared Evaluation Measurement Tools

Institutions Two and Three employed validated IPEC measurement tools to evaluate students’ interprofessional behaviors, including on socialization and valuing [59] at Institution Two, and collaborative practice behavior [60] at Institution Three. Institutions Two and Five administered Robey’s Self-Attributions Scale [61] to assess students’ self-perceived skill, comfort, and approach in providing care to individuals with IDDs. Reporting was limited to eight academic terms at Institution Two and one academic term at Institution Three.

### 4.4. Qualitative Reflections

Qualitative reflections reinforced quantitative findings. Students described increased awareness of communication barriers, recognition of ableism within clinical environments, and greater confidence in adapting care for individuals with IDDs. Many also reported that the experiences challenged preconceived assumptions and strengthened their readiness to engage in collaborative, person-centered care. In the simulation experience at Institution Five, some students reported that the experience was their first experience interacting with someone with IDDs [70]. Advanced practice residency learners additionally expressed preparedness to implement organizational improvements, including changes to workflow, communication practices, accessibility, and staff preparation [78]. See Table 2 for descriptions of programs across partner institutions. Institutional demographic characteristics for each partner institution are provided in the table to contextualize program implementation and participant populations.

## 5. Discussion

The IDD Health Equity Consortium built on, but was distinct from, other IDD-inclusive health-professional education efforts, including ones developed through UCEDD and LEND programs. Unlike UCEDD and LEND programs, that provided long-standing, federally funded training within individual institutions, this initiative was, to our knowledge, the first ACL-funded effort to require formation of a national consortium. The IDD Health Equity Consortium was unique as a multi-institutional and cross-sector collaborative effort. The use of the Collective Impact Model enabled coordinated development of a flexible suite of educational materials and practice experiences that no single institution could have achieved independently. Dissemination of developed programs extended beyond the original five partner institutions early in the implementation phase of the Collective Impact Model. In this early period of the phase of sustaining action and impact through dissemination, we did not have sufficient data to determine whether adoption was equitable across institution types, including under-resourced institutions, highlighting the need for future evaluation of uptake and access.

IDD Health Equity programs demonstrated the feasibility and early impact of applying the Collective Impact Model to advance IDD-inclusive health care professional education across multiple institutions. The meaningful involvement of individuals with IDDs as co-designers, advisors, and simulated patients represented a significant shift toward authentic inclusion and enhanced the relevance of training experiences. Several programs, including the SOI-IHF program and simulation programs developed at Institutions Three and Five, were made publicly available, with additional resources forthcoming. Uptake of these materials by more than 100 institutions reflected growing national demand for IDD-inclusive resources for education of health care professionals.

A major contribution of the IDD Health Equity program was the creation of Communication, Measurement, and Education Practice and Policy Consortium Action Networks. These action networks functioned as the operational mechanism through which Collective Impact Model principles were enacted to enable participants from multiple institutions and stakeholder groups to exchange expertise, align priorities, develop evaluation strategies, and disseminate educational innovations beyond their originating institutions.

The Consortium made progress toward sharing leadership and implementing the five tenets of the Collective Impact Model. Efforts to share leadership, establish a common agenda, and provide backbone support were ongoing and facilitated coordination and communication, though some issues may have remained. Because the IDD Health Equity Consortium involved multi-institutional, cross-sector collaborations and brought together organizations with different contexts, strengths, resources, and priorities, some differences in perspectives and approaches were expected and were considered useful when discussing and developing multiple aligned programs. Future consortia should invest early in structured processes for sharing leadership, aligning priorities within a common agenda, and strengthening backbone support to manage expected differences and use them to advance collective impact.

Shared measurement proved difficult to operationalize, in part because partner institutions varied in their evaluation capacity and in their use of competency frameworks. This included uneven adoption of the ADHCE Core Competencies and IPEC competencies during program development, as well as differences in institutional priorities, staffing, and data-collection infrastructure. These contextual variations limited the development of common metrics and reduced comparability across sites. The partial success in achieving shared measurement highlighted the need for ongoing evaluation, adaptation, and capacity building for organizations seeking to implement the Collective Impact Model. Anticipating such challenges may require early alignment on competency frameworks, early alignment on shared evaluation measures, and planned support for institutions with limited evaluation resources.

Early-stage evaluations relied on self-report measures, introducing the possibility of social desirability bias in participant responses. Moreover, some observed improvements may have reflected the broader institutional cultures of partnering institutions, that already emphasized interprofessional collaboration and IDD-inclusive practices. Such contextual factors made it challenging to isolate the specific contribution of Consortium programming and represented threat to validity; however, initial findings suggested potential for more rigorous empirical evaluation of Consortium initiatives. Plans are underway to examine downstream clinical and institutional outcomes in acute care settings where Consortium-trained students complete clinical experiences. This approach could enable assessment of whether differences emerge in practice outcomes and clinical outcomes compared with settings that have not hosted Consortium-trained learners. Yet, even if improvements at the practice and/or clinical level are identified, the assumption that gains in learners’ knowledge, skills, and attitudes translate into improved outcomes, and that coordinated multi-institutional action accelerate change, would be theoretically grounded but empirically difficult to substantiate. Establishing causal relationships within complex educational and organizational environments presents inherent methodological challenges.

Finally, the Consortium’s ability to secure supplemental funding at several partner institutions accelerated program development, dissemination, and evaluation, but also highlighted disparities in institutional capacity that may limit future scalability in less-resourced settings. Sustaining essential backbone functions, such as coordination, communication, and compensated advocate involvement, may be difficult without ongoing external support.

## 6. Conclusions

Even with these limitations, IDD Health Equity programs established an important foundation for building a more IDD-inclusive prepared workforce and advancing interprofessional, IDD-competent care. The initiative demonstrated a replicable model for integrating IDD-inclusive content into health-professional education and showed the potential of coordinated multi-institutional, cross-sector collaboration. Future work must identify strategies to support adaptation and sustainability across diverse institutional environments and develop standardized, scalable evaluation methods. Strengthening shared measurement and testing the causal pathways to practice and clinical outcomes will be essential.

## Figures and Tables

**Table 1:** Collective Impact phases, dates, and aligned activities with tenets.

Phases	Months and Program Years	Activities Aligned with Tenets
Phase 1: Generating Ideas and Hosting Dialog	Pre-program: April 2020–August 2020	Activities to complete included: Formed IDD Health Equity Consortium (Common Agenda). Joint decision—applied for and obtained funding (Common Agenda).
Phase 2: Initiating Action	Year one: September 2020–August 2021	Activities included: Completed training in Collective Impact Model (FSG) (Common Agenda). Completed initial discussions intersectionality (Common agenda). Began scoping review of literature (Common agenda). Adopted communication protocol (Continuous Communication). Launched newsletter (Continuous Communication). Hired Program Coordinator and assistants (Backbone Support). Formed Steering Committee and Action Networks (Backbone Support). Consortium Action Networks organized and began meeting (Common Agenda, Mutually Reinforcing Activities, Shared Measurement, Continuous Communication).
Phase 3: Organizing for Impact	Year two: September 2021–August 2022	PPDM process to identify shared themes (Common Agenda). Formation of Advocate Advisory Committee (Backbone Support). Development of website and social media platforms and strategies (Continuous Communication). Measurement Action Network meetings begin (Shared Measurement). Development of social media strategy (Continuous Communication). Establishment learning management system sites spaces, simulation spaces, faculty time (Backbone Support).
Phase 4: Beginning Implementation	Years three and four: September 2022–August 2024	Implementation of programs at partner institutions (Mutually Reinforcing Activities). Program-specific evaluation measures developed (Shared Measurement). Dissemination of programs to additional institutions begun (Mutually Reinforcing Activities).
Phase 5: Sustaining Action and Impact	Year five and extension: September 2024—completion of grant	Funding secured for implementation of AISLE program at other institutions (Backbone Support). Educational materials and practice experiences became publicly available (Mutually Reinforcing Activities).

**Table 2:** Descriptions of programs across partner institutions, evaluation measures, and institutional demographics.

Institution	One (public institution Midwest US city	Two (private institutions Midwest US city)	Three (public institution Midwest US city	Four (private northern US city)	Five (mid Atlantic city)
Type of learning	Focus on advocacy and community-supports	Didactic, Service-learning	Simulation—IPE	Didactic	Simulation—IPE
Description	Led PPDM process [17,55]. Assisted development and conducted evaluation pilot of 5-module Inclusive Health Fundamentals [67] for professionals, students, lay public.	Pilot and ongoing 5-module Inclusive Health Fundamentals; 10-week program with 9 didactic modules and 3 telehealth sessions with person supported by staff to develop and f/u on use of wellness goal with action plan [62,72].	Individuals with IDD (IWIDD) and family members as standardized patients [65].	Three programs 5-module Inclusive Health Fundamentals [67]. 13-module program for acute care nurses in three hospital systems [68]. Advanced Practice Providers Residency program [32].	2 h virtual simulation developed for institutions with limited access to interprofessional students to participate. Included 7 pre-simulation instructional modules and case scenarios to be discussed by participating students. Included participation of IWIDD as “expert patient” [70].
Competencies					
ADHCE [10]	No	Yes	Yes	No	Yes
IPEC [58]	No	Yes	Yes	No	Yes
Involvement design, implementation, evaluation	Yes, IWIDD and family	Yes, IWIDD and staff caregivers.	Yes, IWIDD and family.	Yes, IWIDD and family	Yes, IWIDD and family.
Offered	Assisted in development and pilot of SOI-IHF in 2023.	SOI-IHF ongoing since 2023. AISLE—ongoing since Fall 2022.	Simulation ongoing since Fall 2022.	SOI-IHF ongoing 2023 Acute care nursing program ongoing Advanced Practice Providers Residency program ongoing.	Simulation ongoing since spring 2023.
Virtual experiences	Virtual	Virtual	In person and virtual	Virtual	In person and virtual.
Number of students	586 students total in pilot. 441 completed pre and 412 post-evaluation.	SOI-IHF ≈ 300 students. AISLE-161 over 8 terms partnered with 55 indivisuals with IDDs receiving residential services.	≈90 per term/2 terms per year.	110 Acute care nurses Advanced Practice Providers Residency program (not reported).	≈500 with most recent simulation (conducted March 2026).
Interprofessional	22 participants from affected groups participated in PPDM.	Students from 11 professional programs [62,72].	Medical, Dental, and Nurse Practitioner students from two institutions [65].	Multiple disciplines and lay personnel.	Nursing, Occupational Therapy, Physical therapy, Pharmacy, Physician Assistant students from 3 institutions [70].
Evaluation	Developed themes for program development and evaluation from PPDM process. Conducted evaluation pilot of SOI-IHF. Knowledge improved. Confidence in communication improved.	Pre–post Robey’s scale [61]. ISVS-9 [59]. Each telehealth visit student and faculty completed interprofessional process of care [74] and telehealth survey [73].	Pre–post IPECC–SET27 [60] (n = 136, *p* < 0.0001).	Pre–post surveys. Follow-up at sites.	Pre–post Robey’s scale [61].
Nursing students Other	Not available.	50% 50% [62,72].	Not available.	Not available.	66% 33% PT and OT students [70].
Demographics of institutions					
Male	45%	21%	46.6%	13.4%	27.3%
Female	55%	78%	53.4%	86.6%	72.2%
White	62.8%	55%	22%%	63.0%	67.0%
Black	8.4%	20%	8%	10.0%	6.5%
Hispanic	6.3%	20%	36%	9.0%	10.7%
Asian	11.2%	19%	21%	9.3%	7.8%
Other	10.4% [79]	<2% [80]	5% [81]	8.0% [82]	4.5% [83]

## Data Availability

The evaluation data for the IDD Health Equity workforce development initiative were obtained from course evaluations and program assessments conducted by partnering institutions. The data were collected for administrative and quality-improvement purposes and may contain student information protected under US federal law. Because each dataset is maintained by its respective institution and subject to institutional privacy policies, the data are not publicly available. De-identified data may be provided by the authors upon reasonable request and with appropriate institutional approvals. Program materials are publicly available or are available upon request from authors.
